# An IoT-based low-cost architecture for smart libraries using SDN

**DOI:** 10.1038/s41598-024-57484-2

**Published:** 2024-03-25

**Authors:** Hui Xu, Wei-dong Liu, Lu Li, Qi Zhou

**Affiliations:** 1 Heilongjiang University of Chinese Medicine, Harbin, 150040 People’s Republic of China; 2 Heilongjiang Provincial Big Data Center of Government Affairs, Harbin, 150028 People’s Republic of China; 3 Harbin University of Science and Technology, Harbin, 15006 People’s Republic of China; 4 North China Electric Power University, Library, Beijing, 102206 People’s Republic of China

**Keywords:** Internet of Things, Smart libraries, Software-defined networking, Radio-frequency identification, Computational science, Computer science, Information technology

## Abstract

In the evolving landscape of smart libraries, this research pioneers an IoT-based low-cost architecture utilizing Software-Defined Networking (SDN). The increasing demand for more efficient and economical solutions in library management, particularly in the realm of RFID-based processes such as authentication, property circulation, and book loans, underscores the significance of this study. Leveraging the collaborative potential of IoT and SDN technologies, our proposed system introduces a fresh perspective to tackle these challenges and advance intelligent library management. In response to the evolving landscape of smart libraries, our research presents an Internet of Things (IoT)-based low-cost architecture utilizing SDN. The exploration of this architectural paradigm arises from a recognized gap in the existing literature, pointing towards the necessity for more efficient and cost-effective solutions in managing library processes. Our proposed algorithm integrates IoT and SDN technologies to intelligently oversee various library activities, specifically targeting RFID-based processes such as authentication, property circulation management, and book loan management. The system's architecture, encompasses components like the data center, SDN controllers, RFID tags, tag readers, and other network sensors. By leveraging the synergy between RFID and SDN, our innovative approach reduces the need for constant operator supervision in libraries. The scalability and software-oriented nature of the architecture cater to extensive library environments. Our study includes a two-phase investigation, combining practical implementation in a small-scale library with a simulation environment using MATLAB 2021. This research not only fills a crucial gap in current knowledge but also lays the foundation for future advancements in the integration of IoT and SDN technologies for intelligent library management.

## Introduction

The importance of an IoT-based low-cost architecture lies in its potential to democratize and extend the benefits of the IoT to a broader range of applications and users^1,2^. By focusing on cost-effectiveness, such architectures enable the deployment of IoT solutions in diverse settings, including resource-constrained environments and developing regions. This affordability facilitates widespread adoption in sectors such as agriculture, healthcare, smart cities, and industrial automation, where cost-efficient connectivity and data exchange are paramount^3^. The low-cost IoT architecture can empower businesses, communities, and individuals to harness the transformative capabilities of IoT technology, fostering innovation, efficiency, and improved quality of life across various domains^4^.

Smart libraries play a crucial role in modernizing and enhancing traditional library services through the integration of advanced technologies. The importance of smart libraries lies in their ability to leverage innovations such as IoT, artificial intelligence, and data analytics to create more efficient and user-centric library experiences^5^. These technologies facilitate tasks like automated cataloging, personalized recommendations, and real-time tracking of library resources^6^. Smart libraries enhance accessibility to information, offering users a seamless and interactive environment for research and learning. Additionally, they contribute to the preservation of valuable resources by employing digital archiving and conservation methods^7^. The application of smart libraries spans across educational institutions, research facilities, and public spaces, promoting the evolution of libraries into dynamic hubs that adapt to contemporary information needs and technological advancements^8^.

SDN holds paramount importance in revolutionizing traditional network architectures by providing a more flexible, scalable, and programmable approach to network management. By decoupling the control plane from the data plane, SDN allows for centralized control, enabling administrators to dynamically allocate network resources and implement changes efficiently^9^. The significance of SDN lies in its ability to streamline network provisioning, enhance scalability, and simplify network management, leading to improved operational efficiency^10^. SDN finds applications across various sectors, including data centers, telecommunications, and enterprise networks. In data centers, SDN facilitates the orchestration of resources and ensures optimal traffic flow, contributing to better overall performance. In telecommunications, SDN enables the creation of agile and programmable networks, paving the way for innovations like 5G^11^. Enterprises benefit from SDN by achieving greater control over their network infrastructure, supporting dynamic business requirements, and enhancing security through centralized policy management. Overall, SDN plays a pivotal role in reshaping network architectures to meet the demands of modern, dynamic, and data-intensive applications^12^.

The IoT-based low-cost architecture for smart libraries utilizing SDN is of significant importance in transforming traditional libraries into intelligent, connected spaces^13^. This innovative approach leverages the IoT to enhance library services, improve resource management, and provide an enriched experience for users. By integrating sensors, RFID technology, and other IoT devices, the system enables real-time monitoring of book availability, user preferences, and environmental conditions within the library^14^. SDN comes into play by offering a centralized and programmable network infrastructure, allowing efficient communication and control of diverse IoT devices^4^. This architecture not only optimizes resource allocation and energy efficiency but also enhances security through centralized monitoring. Applications of this IoT-based low-cost architecture for smart libraries are diverse, including smart inventory management, personalized user services, and data-driven decision-making for library administrators^15^. Overall, this integrated approach transforms traditional libraries into dynamic, responsive, and technology-driven hubs that cater to the evolving needs of library users in the digital age^16^.

An IoT-based low-cost architecture is a technological framework designed to integrate the IoT into a system or environment with a primary focus on cost-effectiveness^17^. This architecture utilizes IoT devices, which are interconnected physical devices equipped with sensors, actuators, and communication modules, to efficiently collect and exchange data^18^. Emphasizing affordability, it enables the deployment of IoT applications in various scenarios with budget constraints, optimizing resource utilization and ensuring efficient connectivity among IoT devices. The goal is to bring the benefits of IoT, such as real-time data monitoring and enhanced automation, to environments where cost efficiency is paramount. Smart libraries, on the other hand, are modernized library systems incorporating advanced technologies like IoT, artificial intelligence, and data analytics to streamline operations and services^19^. These libraries use automation for tasks such as cataloging, check-in/check-out, and inventory management, enhancing efficiency and accuracy. Featuring digitized collections, interactive learning spaces, and personalized services, smart libraries create an intelligent and user-centric environment, adapting to the evolving needs of patrons in the digital age^20^.

SDN, or Software-Defined Networking, is a revolutionary networking paradigm that revolutionizes traditional network architectures by separating the control plane from the data plane^21^. In SDN, network intelligence and decision-making are centralized through a software-based controller, offering programmability, flexibility, and dynamic management of network resources. This decoupling enables efficient network configuration, monitoring, and optimization through software applications, fostering automation and agility in response to changing network conditions^22^. The goal is to simplify network management, enhance scalability, and expedite the deployment of new services. When applied to an IoT-based low-cost architecture for smart libraries, SDN becomes instrumental in creating intelligent and affordable library systems^23^. By integrating IoT devices like sensors and smart devices into the library environment, data collection and diverse applications are enabled. The emphasis on being low-cost ensures accessibility for libraries facing resource constraints, with SDN providing a programmable and centralized control plane for dynamic adaptation to changing requirements. This integrated approach enhances library services, facilitates real-time monitoring, and supports innovative applications, ultimately establishing a smart and interconnected library ecosystem^24^.

Our research paper significantly contributes to the field of smart libraries by introducing a groundbreaking IoT-based low-cost architecture. Unlike prior studies that have primarily focused on IoT-based low-cost architecture, our work explores a novel approach by integrating IoT and SDN technologies to optimize library activities. The key contribution lies in adapting the synergy of IoT and SDN, originally designed for smart libraries. Our proposed algorithm, leveraging RFID-based processes and SDN-based network configuration, provides a unique and efficient strategy for enhancing library intelligence. The integration of RFID technology for authentication, property circulation management, and library book loan management, along with SDN's streamlined communication infrastructure, forms a pioneering architecture. This novel framework not only reduces the need for constant operator supervision in libraries but also presents a scalable and software-oriented solution. The hierarchical tree topology, formed through our clustering algorithm, further enhances communication efficiency, offering a valuable and innovative perspective for future developments in IoT-based library systems.

- **Innovative IoT-Based Architecture:** Introduces a groundbreaking low-cost architecture integrating IoT and SDN technologies.
- **Comprehensive RFID-Based Processes:** Proposes an algorithm for authentication, property circulation management, and library book loan management using RFID.
- **Efficient SDN-Based Network Configuration:** Presents a novel approach to SDN-based network configuration and routing.
- **Reduced Operator Dependency:** Emphasizes the reduction of constant operator supervision in overseeing library processes.
- **Cost-Effective Strategy:** Highlights the cost-effectiveness of the proposed architecture.
- **Hierarchical Tree Topology:** Introduces a hierarchical tree topology through a clustering algorithm.
- **Scalability and Adaptability:** Offers a scalable and adaptable framework for resource management.
- **Future Research Directions:** Identifies prospective tasks for future investigations in the integration of IoT and SDN technologies.

## Related works

Considering related works in the field of an IoT-based low-cost architecture for smart libraries using SDN is crucial for several reasons. Firstly, it allows researchers and practitioners to build upon existing knowledge and identify gaps in current solutions, ensuring that the proposed architecture addresses specific challenges in the context of smart libraries. Secondly, a review of related works facilitates the incorporation of successful strategies and lessons learned from prior implementations, enhancing the likelihood of success and efficiency in the development of the proposed architecture. Lastly, a comprehensive understanding of the existing literature helps in creating a more innovative and contextually relevant solution, positioning the IoT-based architecture within the broader landscape of smart libraries and SDN applications. In this regard, Also, Sankar, Ramasubbareddy^25^ introduced a routing protocol, CT-RPL, designed specifically for the IoT applications. The protocol is based on a cluster tree structure, aiming to optimize energy efficiency and maximize the overall network lifetime. By organizing nodes into clusters and establishing a hierarchical tree topology, CT-RPL efficiently manages communication and routing within the IoT network. The key focus of the protocol is on prolonging the lifespan of IoT devices by minimizing energy consumption, thus addressing a critical challenge in IoT deployments. The paper provided insights into the design, implementation, and performance evaluation of CT-RPL as a promising solution for enhancing the longevity of IoT networks.

Luo^6^ presented an approach to enhancing the security of IoT environments. The proposed system leveraged SDN to create a distributed intrusion detection system tailored for IoT devices. By employing optimized forests, the system aimed to efficiently detect and mitigate potential security threats across a decentralized network. The utilization of SDN provided a centralized control plane, allowing for dynamic and programmable management of network resources, which is particularly advantageous for securing diverse and interconnected IoT ecosystems. The paper contributed to the field by addressing the unique security challenges posed by IoT devices through a distributed SDN-based approach with optimized forests for effective intrusion detection.

In addition, Gupta, Juneja^26^ proposed a method to address the challenges of network resource management in the context of 5G-enabled IoT applications for smart healthcare. The authors introduced an intelligent technique that aims to optimize the allocation and utilization of network resources to enhance the performance and efficiency of healthcare applications. The study involved the integration of artificial intelligence or machine learning methods to analyze and dynamically manage network resources, ensuring the seamless operation of 5G-IoT applications in the healthcare domain. The proposed approach contributed valuable insights into improving the reliability and responsiveness of smart healthcare systems, fostering advancements in the integration of 5G and IoT technologies for healthcare applications.

As well, Bhuiyan, Billah^27^ focused on the development of a practical and applicable model for healthcare monitoring in both rural and urban settings using the IoT. The proposed system leveraged IoT technologies to create a ubiquitous healthcare monitoring infrastructure, ensuring that individuals in diverse geographical areas can access healthcare services seamlessly. The model considered the specific challenges and requirements of both rural and urban environments, aiming to provide an inclusive and effective healthcare monitoring solution. This paper contributed to the advancement of IoT-based healthcare systems by offering a feasible and adaptable model tailored to the unique characteristics of different regions.

Besides, de Melo, Miani^28^ addressed the critical issue of securing home networks through the introduction of the FamilyGuard security architecture. The proposed model focused on anomaly detection within home environments, aiming to safeguard the interconnected devices and systems commonly found in modern households. The authors presented a comprehensive approach to network security, utilizing anomaly detection techniques to identify potential threats or irregularities. By addressing the unique challenges posed by home networks, FamilyGuard offered a tailored and effective security solution to protect the privacy and integrity of users' connected devices within a domestic setting.

Additionally, Elhoseny, Siraj^29^ concentrated on addressing the challenges of energy efficiency and security in IoT applications. The authors proposed a mobile agent-based protocol designed to enhance the sustainability of IoT systems. The protocol aimed to optimize energy consumption in IoT devices while ensuring robust security measures. By employing mobile agents that can autonomously move between devices to perform specific tasks, the protocol seeks to minimize energy usage, prolong device lifespan, and enhance overall system efficiency. Additionally, the authors emphasized the importance of securing IoT applications against potential threats, contributing to the development of sustainable and secure IoT solutions. Table 1 indicated specification of investigated related works.

**Table 1:** Related works.

Authors	Main Idea	Advantages	Disadvantages	Simulation Environment	Dataset
Sankar, Ramasubbareddy^25^	Introducing a routing protocol, CT-RPL, designed specifically for the IoT applications	High fault tolerant High scalability	Poor adaptability	Contiki COOJA	100 routers
Luo^6^	Presenting an approach to enhancing the security of IoT environments	Poor complexity	Poor scalability Poor security	MATLAB	1,074,992 samples
Gupta, Juneja^26^	Proposed a method to enhancing the security of IoT environments	High interpretability	Poor adaptability	Python/ Keras	32 k records
Bhuiyan, Billah^27^	Developing of a practical and applicable model for healthcare monitoring in both rural and urban settings	High security High privacy	Poor adaptability	Java	10 patient’s sample data
de Melo, Miani^28^	Addressing the critical issue of securing home networks through the introduction of the FamilyGuard security architecture	High adaptability High safety	Poor generalizability	Raspberry Pi	78,836 samples
Elhoseny, Siraj^29^	Concentrating on addressing the challenges of energy efficiency and security in IoT applications	High flexibility High adaptability	Poor security Poor robustness	Soft-EdgeNet/ MADIT	100 samples
Chiliquinga, Manzano^22^	Exploring an approach for monitoring IoT networks by leveraging SDN and efficient traffic signatures	High flexibility High adaptability	Poor generalizability	Python	26 samples
Njah, Pham^30^	Proposing an innovative approach to flow management in a smart digital campus using SDN	High scalability	High complexity Poor security Poor reliability	Julia/ SNDlib library	25 nodes
Gordon, Batula^31^	Presenting an innovative approach to enhancing the security of smart homes	High resiliency High security	Poor interpretability Poor scalability	–	3,066,585 packets
Ganesan, Hwang^32^	Presenting an approach to network traffic classification in a SDN-enabled Fiber-Wireless-Internet of Things (FiWi-IoT) smart environment	High security High adaptability	Poor scalability High complexity Overhead	Orange-ML	28 devices
Wang and Wang^33^	Exploring a strategic approach to mitigating Distributed Denial-of-Service (DDoS) attacks within SDN environments	High resiliency High scalability	Overhead High complexity Poor adaptability	Mininet	2400 samples
Abid, Afaqui^34^	Exploring the transformative journey of the IoT towards becoming smarter and more adaptive through the integration of SDN principles	High scalability High flexibility Poor complexity	Poor security Poor interpretability	DPWSim	34 samples
Ours	Imbuing libraries with intelligence leverages the synergy of IoT and SDN technologies	High scalability Cost-effective Low latency	Poor integrity	MATLAB	

As well, Chiliquinga, Manzano^22^ explored an approach for monitoring IoT networks by leveraging SDN and efficient traffic signatures. The proposed method aimed to address the challenges associated with the growing complexity and diversity of IoT devices and their communication patterns. By incorporating SDN, the network monitoring process becomes more flexible and dynamic, allowing for adaptive responses to emerging threats or changes in network behavior. Additionally, the use of cost-effective traffic signatures helped in efficiently identifying and analyzing IoT-related traffic, optimizing resource utilization. The paper emphasized a progressive monitoring strategy that evolves with the evolving landscape of IoT networks, enhancing overall network security and performance.

Also, Njah, Pham^30^ proposed an innovative approach to flow management in a smart digital campus using SDN. The proposed scheme focused on efficiently managing network flows by considering both service requirements and available resources. By leveraging the programmability and central control offered by SDN, the system aimed to dynamically adapt to the varying demands of services within a digital campus. This approach enhanced the overall performance and resource utilization of the network infrastructure, ensuring that services in the smart campus environment are delivered optimally. The paper contributed to the advancement of SDN-based solutions tailored for complex digital campus scenarios, emphasizing the importance of balancing service requirements and resource management for effective flow control.

Furthermore, Gordon, Batula^31^ presented an innovative approach to enhancing the security of smart homes. The authors proposed a solution that integrates SDN with low-cost traffic classification techniques. This combination allowed for dynamic and programmable control over the home network, enabling adaptive responses to security threats. The use of low-cost traffic classification enhanced the efficiency of identifying and managing different types of network traffic associated with smart home devices. By leveraging SDN's capabilities and cost-effective traffic classification, the paper contributed to the development of practical and accessible security measures tailored for the unique challenges presented by smart home environments, ultimately aiming to provide homeowners with a more resilient and responsive defense against potential cybersecurity threats.

Moreover, Ganesan, Hwang^32^ presented an approach to network traffic classification in a SDN-enabled Fiber-Wireless-Internet of Things (FiWi-IoT) smart environment. The study employed supervised machine learning (ML) models to classify network traffic efficiently. By integrating SDN with FiWi-IoT infrastructure, the proposed method allowed for centralized and programmable control, enhancing adaptability to dynamic network conditions. The application of supervised ML models provided an intelligent mechanism for accurately categorizing diverse traffic types within the smart environment. This approach contributed to improved network management, ensuring that the FiWi-IoT network is optimized for various traffic patterns and enhancing the overall efficiency and responsiveness of the smart environment.

And, Wang and Wang^33^ explored a strategic approach to mitigating Distributed Denial-of-Service (DDoS) attacks within SDN environments. The study focused on developing an efficient and cost-effective defense mechanism against DDoS threats, leveraging the programmability and centralized control capabilities inherent in SDN. By employing intelligent traffic monitoring and analysis, the proposed method identified and mitigated malicious traffic patterns, thereby enhancing the network's resilience to DDoS attacks. The emphasis on efficiency and low-cost solutions is crucial for making the defense mechanisms accessible and practical, addressing the economic considerations associated with implementing robust security measures in SDN-based networks.

Additionally, Abid, Afaqui^34^ explored the transformative journey of the IoT towards becoming smarter and more adaptive through the integration of SDN principles. The study delved into the evolution of IoT technologies, emphasizing the need for a software-defined approach to address the increasing complexity, heterogeneity, and dynamic nature of IoT environments. By adopting SDN, the proposed evolution sought to enhance the intelligence and flexibility of IoT networks, allowing for efficient resource management, dynamic adaptation to changing conditions, and improved overall performance. The paper discussed key concepts and challenges associated with this evolution, providing insights into the future of smart and software-defined IoT systems.

## Proposed algorithm

The proposed method for imbuing libraries with intelligence leverages the synergy of IoT and SDN technologies. This architecture demonstrates the capability to intelligently oversee a diverse array of library activities, thereby mitigating the requirement for constant operator supervision. The suggested framework incorporates SDN to streamline the intricacies associated with resource management in IoT, offering an efficient and cost-effective strategy. The supported activities within libraries employing this architecture include:

1. RFID-based processes encompass:

- Authentication
- Property circulation management
- Library book loan management

2. Data exchange based on IoT SDN

**Figure 1:**
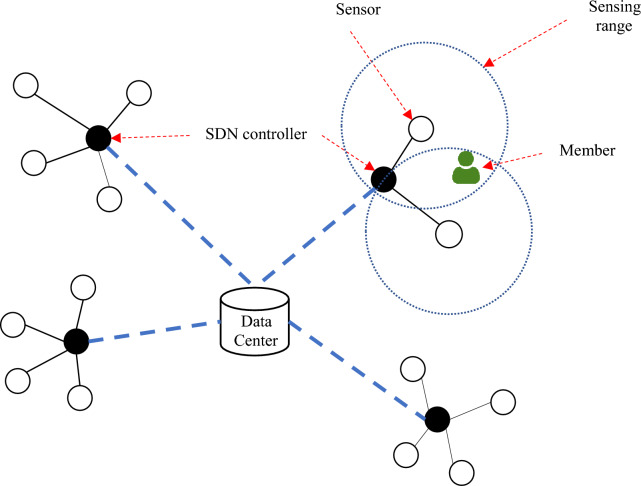
A view of the proposed smart library model.

Thus, the entire intelligent processes in the library can be categorized into two groups based on RFID and SDN, depending on the platform used. In the proposed architecture, authentication, property circulation management, and book lending processes utilize RFID technology and operate on the IoT communication platform. Conversely, the information exchange mechanism relies on SDN architecture to furnish an efficient communication platform in smart libraries. Figure 1 illustrates the proposed architecture, which, based on the amalgamation of Internet of Things and software-oriented network technologies, can be segmented into the following fundamental components:

- Data center
- SDN controllers
- RFID tags and tag readers, along with other network sensors
- Users

The architecture presented in Fig. 1 offers the potential to modernize key processes in traditional libraries. In this proposed architecture, intelligent library information management relies on a data center, serving as the hub for organizing crucial data for intelligent library management. The data center accommodates key information in the following tables:

- Books information
- Member information
- Book loan information

The table for book information encompasses key attributes associated with each book in the library. Within this table, access to individual books is facilitated through a master key. This unique key, recorded in the book information table, is also stored in the RFID tag affixed to the corresponding book. Consequently, by scanning the RFID tag and extracting its unique code, the book's information can be retrieved via the data center. Similarly, the member information table comprises distinct characteristics of each library member, with each member differentiated by a unique membership ID. This unique code is stored in the RFID tag on the member's card, enabling the retrieval of all personal information through tag access. The book loan information table serves as a communication interface between the aforementioned tables, managing transactions related to book entries and exits. Each entry in this table describes the loan or return of a book by a member, utilizing foreign keys corresponding to the unique identifiers of the book and the member. Additionally, it includes time information regarding the book's loan and return. Two additional tables are employed to oversee book reservation processes and library property management. These tables mirror the structure of the book loan information table, storing information on book reservations or transfers of library property by members. It is evident that components such as book and member RFID tags, the data center, and RFID tag reader nodes play essential roles in processes related to member authentication, book loans, and returns.

Conducting the mentioned processes intelligently, especially in extensive libraries, necessitates the utilization of an efficient communication infrastructure. The proposed method addresses this requirement through the integration of IoT and SDN, as illustrated in Fig. 1. The suggested architecture categorizes the array of smart library sensors using SDN technology, organizing each group of adjacent sensors into a subnet. Controller nodes, supervising each sub-network, govern the data exchange between the sensors and the data center within the smart library. This communication mechanism, coupled with RFID technology, offers a solution to concerns related to the model's cost and scalability in expansive environments. The subsequent section provides a detailed description of each process outlined in the proposed smart library model.

### RFID-based processes in the proposed architecture

**Figure 2:**
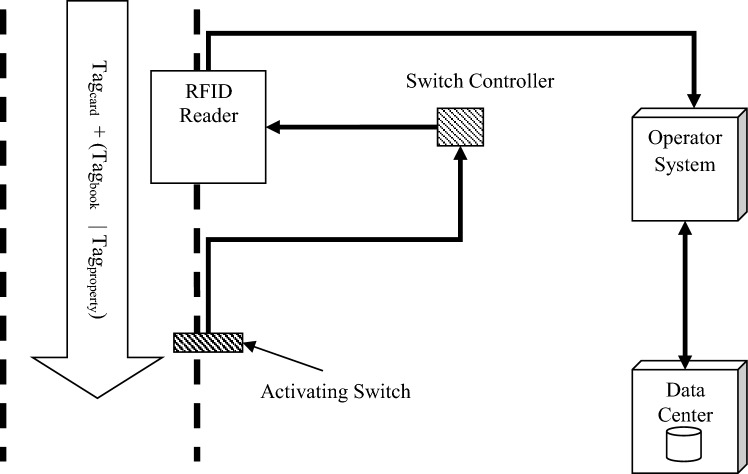
The mechanism of RFID-based processes in the proposed system architecture.

As mentioned, RFID-based processes encompass a range of functions associated with member authentication, property movement control, and library book loan management. In all these processes, three key components—data center, RFID tag, and tag reader—collaborate. Figure 2 illustrates the mechanism of RFID-based processes within the proposed system architecture.

As illustrated in Fig. 2, the architecture of this system comprises four components: tag, tag reader, antenna, and computer network. In the proposed model, passive tags are utilized to minimize the overall implementation cost of the system. This choice is based on the suitable distance of 5–10 m supported by passive tags for effective identification. To detect the presence of a person, a sensor and an activating switch are employed in the identity verification station. When an individual enters the identity verification station, the switch within the sensor triggers the tag reader. The tag reader component is positioned adjacent to the sensor. Consequently, upon switch activation, the tag reader component transmits a coded signal to the tags affixed to the user's membership card, books, or property. Each tag component then emits its unique identification code as a radio signal to the reader.

**Figure 3:**
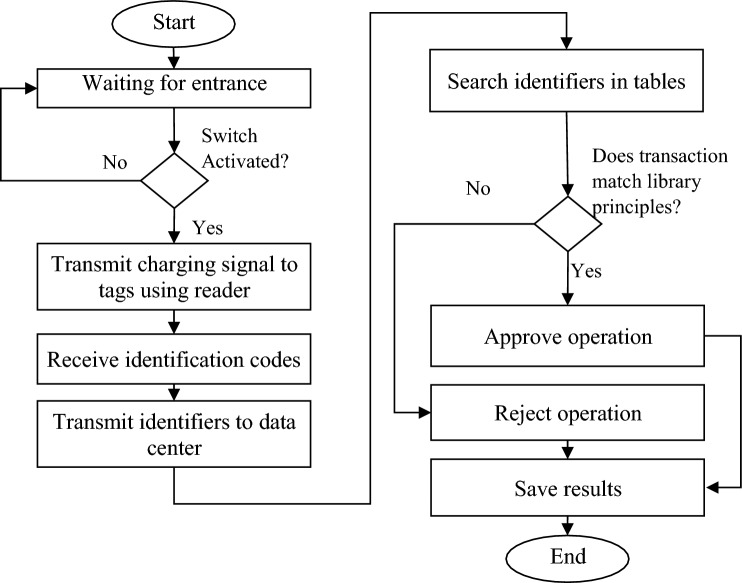
Flowchart of RFID-based processes in the proposed architecture.

Upon receiving the identification codes, this information is transmitted to the computer system linked to the tag reader. Subsequently, the computer system relays this data to the data center via the network. The data center securely stores the encrypted IDs of all members, assets, and books in the library. The received identifier undergoes a search operation within the data center through the tag reader. If the desired identifier is located in the database, a confirmation message is dispatched to the sender's computer system, signaling successful verification. Conversely, if the identifier is deemed invalid, an error message is generated. Upon successful identity verification, the data center records the transaction time and other pertinent details. Notably, the search and validation processes are carried out independently for each member and book/property component. To achieve this, the unique membership ID (Tagcard in Fig. 2) is sought in the member information table, while the book/property's unique ID (Tagbook/Tagproperty) is searched in the books/property information table. The corresponding flowchart depicting these processes is presented in Fig. 3.

**Figure 4:**
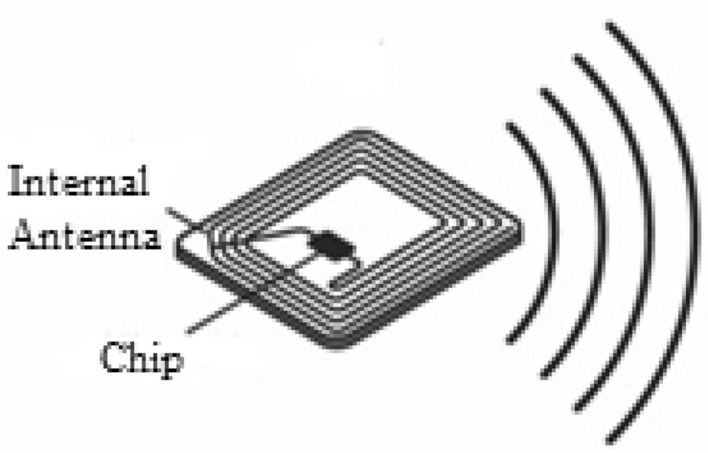
UFH band passive tag structure used in the proposed architecture.

The electronic tags employed to identify library members, books, and assets in the proposed model represent an automated data carrier utilizing RFID technology. The system's architecture adopts passive RFID tags operating in the UHF band, with a reading range spanning 6–10 m. Figure 4 illustrates the structure of these tags. Passive tags, as mentioned, lack an independent energy source for transmitting identifiers to the reader component. Instead, they harness energy from radio frequency pulses dispatched by the reader component. Upon receiving these pulses, the tag charges its internal capacitor, utilizing it as an energy supply source. Subsequently, the tag transmits its information to the reader component through internal antennas. It's noteworthy that the identifiers stored in these specialized electronic tags boast resistance to copying or alteration. The extended lifespan and cost-effectiveness of RFID technology contribute to its advantageous use in creating intelligent libraries.

### SDN-based network configuration and routing

The proposed architecture for smart libraries adopts a scalable and software-oriented network-based mechanism to establish the data exchange pattern among network components. This entails defining the necessary mechanisms for constructing topology and data routing based on that topology—an essential process in the network operations of IoT-based architectures. The topology construction involves creating a network communication infrastructure using a subset of stable communication links. Simultaneously, the routing process dictates how data is exchanged between network sensors (such as members' smartphones, tag readers, and controllers) and the data center, leveraging the established topology. This facilitates data routing for activities like book search, reservation, identity authentication, and other smart library functions between diverse network components. The proposed routing algorithm, detailed in the subsequent section, is a multi-step strategy rooted in the SDN architecture. This algorithm determines optimal routes for data transmission based on a cost function.

#### Construction of topology

The configuration process of the proposed intelligent library architecture commences with the construction and control of the network topology, utilizing a software-based network. In this method, network nodes initially exchange positional information and then establish the clustering structure based on SDN principles. Notably, as a substantial portion of network sensors consists of members' smartphones, all of which are mobile, considering the movement characteristics of these objects during the topology construction process becomes paramount. Therefore, the topology construction process begins by calculating the relative speed between the network nodes. To assess the relative speed of two nodes, the first step involves estimating the distance to the node. In this proposed method, it is assumed that the distance between two nodes can be estimated based on the received signal strength of each node, calculated using the provided equation.

1 $$RSS{I}_{d}={K}_{1}- {K}_{2}{\text{log}}\left(d\right)+ u$$

In the given equation, 'd' denotes the distance between two nodes, and 'u' is an independent random parameter following a Gaussian distribution with a zero mean. Additionally, 'K1' and 'K2' represent the path loss parameters in the 802.11 standard. As expressed in Eq. (1), the distance between two nodes can be approximated through the received signal strength. This relationship can be estimated as described below, without taking the environmental noise factor into account:

2 $${d}_{{c}_{1},{c}_{2}}={e}^{\frac{\left({K}_{1}-rssi\right)}{{K}_{2}}}$$

In the next step of the proposed method, we calculate the relative speed and communication stability. Let's consider two nodes, A and B, at a distance 'd' from each other. These nodes move in a straight line with a speed 'v', and each node has a movement angle 'θ'. Therefore, the velocity vector for nodes A and B will be denoted as $\overline{{V }_{A}}=\left({v}_{A}, {\theta }_{A}\right)$ and $\overline{{V }_{B}}=({v}_{B}, {\theta }_{B})$. We assume that the radio range of nodes is 'R', and.

d < R. The relative velocity vector of the two nodes can be calculated using the following equation:

3 $$\overline{{V }_{BA}}=\overline{{V }_{B}}-\overline{{V }_{A}}=({v}_{BA},{\theta }_{BA})$$

In the above relationship, θ_BA is the relative angle of two nodes A and B. also:

4 $${v}_{BA}=\sqrt{{\left({v}_{B}cos{\theta }_{B}- {v}_{A}cos{\theta }_{A}\right)}^{2}+{\left({v}_{B}sin{\theta }_{B}- {v}_{A}sin{\theta }_{A}\right)}^{2} } ,$$

5 $${\theta }_{BA}={{\text{tan}}}^{-1}\frac{{v}_{B}sin{\theta }_{B}- {v}_{A}sin{\theta }_{A}}{{v}_{B}cos{\theta }_{B}- {v}_{A}cos{\theta }_{A}}$$

Using relations (4) and (5), it can be shown that the duration of two nodes being neighbors will be equal to:

6 $${T}_{neighbor}=\frac{dcos{\theta }_{BA}+ \sqrt{{r}^{2}-{d}^{2}{{\text{sin}}}^{2}{\theta }_{BA}}}{{v}_{BA}}$$

Using the above relationship, it is possible to predict whether two nodes A and B will be neighbors after the time interval tΔ or not? This will happen if T_neighbor > ∆t. In this case, the similarity of the movement pattern of two users A and B will be stored in a matrix as follows:

7 $${T}_{A,B}=\left| lo{c}_{a}^{current}- lo{c}_{b}^{current}\right|+\left| lo{c}_{a}^{future}- lo{c}_{b}^{future}\right|$$

In the above relationship, loc_a^current is the current position of node A as (x_a,y_a) and loc_a^future is the predicted position for node A after the time interval tΔ and is calculated as follows:

8 $$lo{c}_{a}^{future}=({x}_{a}+ {V}_{{x}_{a}}* \Delta t , {y}_{a}+ {V}_{{y}_{a}}* \Delta t )$$

In the above relationship, ${V}_{{x}_{a}}$ represents the speed of the node along the x-axis, and ${V}_{{y}_{a}}$ is its speed along the y-axis. By calculating the value of T_ab_ for each pair of nodes in the network, a portion of the similarity matrix is formed. This matrix contains the degree of similarity in the movement patterns of each pair of nodes. All nodes transmit their matrix portions to the data center node, contributing to the construction of the topology based on the clustering structure. The central node responsible for clustering the moving nodes integrates these ${T}_{ab}$ matrix parts and categorizes nodes into clusters using two fundamental rules. In this method, nodes with similar movement patterns are grouped into a cluster. To assess the similarity of the movement pattern between two nodes, the following conditions are checked:

1. The term "two nodes should be in the same radio range (both nodes have direct one-step access to each other)" refers to the requirement that two nodes should be within each other's direct radio communication range, enabling a one-step direct connection.
2. Additionally, it should be anticipated that after a period of time Δt, the distance between the two nodes does not exceed a predefined threshold distance.

The second condition involves predicting the persistent position of the connection between two nodes, and based on these criteria, the user's movement pattern information is stored in a matrix, denoted as T. Following these rules, the steps for clustering network nodes by the central node are as follows:

- Input: List of users L and connection period matrix T
- Output: C network clusters
- Repeat the following steps until a node is in the L list.
- Pick a random node x from list L, remove it from L, and create a new cluster in C.
- For each node $y\in L$: if y is a neighbor of x and based on the matrix T, ${T}_{x,y}\ge \Delta t$, then add y to the current cluster in clustering C and omit node y from the list L.
- Go to step 1.

After this process, all network nodes will be grouped into clusters based on their movement patterns, and each cluster will be assigned to the nearest SDN controller node in the form of a subnet. The result will be a set of isolated clusters. At the end of the topology control step, these clusters need to be efficiently connected. The proposed algorithm achieves this by forming the network topology based on an optimal subset of connections for each cluster, facilitated through cluster head controller nodes. In the proposed method, neighbors belonging to other clusters are evaluated for each controller node, and the weight of the connection between two nodes i and j is determined using the provided relationship.

9 $${W}_{i,j}=\frac{|{N}_{(i)}-{B}_{K}^{\prime}|}{|{B}_{K}^{\prime}|}-\frac{P}{{E}_{j}}\times {D}_{ij}$$

In the above relation, ${N}_{(i)}$ represents the neighborhood set of node i. ${B}_{K}^{\prime}$ represents the set of nodes covered by the current topology structure (nodes that are either members of the topology or are located in the neighborhood of at least one of its members). P represents the initial energy of network nodes, and ${E}_{j}$ specifies the current energy of node j. Finally, ${D}_{ij}$ indicates the end-to-end delay in communication between two nodes i and j, which is estimated during the neighbor discovery process.

The weight function used in relation (9) consists of two parts: profit and loss. This function states that connecting two clusters by adding a link between two nodes i and j to the network topology, how much benefit and how much loss will result. The first part of this relationship is $\frac{|{N}_{(i)}-{B}_{K}^{\prime}|}{|{B}_{K}^{\prime}|}$ and it shows the number of new nodes that will be covered by the topology by joining these two clusters. If the selection of a node can add more members to the topology, adding with that node will have a high benefit. On the other hand, if the selection of a node does not add any new member to the topology, then $\frac{|{N}_{(i)}-{B}_{K}^{\prime}|}{|{B}_{K}^{\prime}|}=0$ and relation (9) is negative Will have. As a result, the proposed weight calculation relationship will prevent the addition of ineffective nodes in the topology. The second part of the weight function in relation (9) represents the amount of loss resulting from the selection of node j and is described as $\frac{P{\times D}_{ij}}{{E}_{j}}$. This part of the weight function shows how adding node j to the topology will affect the topology's lifetime and latency.

After evaluating the weight function by the nodes completing the topology (Relation 9), each time the link with the most positive weight is selected and the cluster corresponding to that selection will be added to the topology.

**Figure 5:**
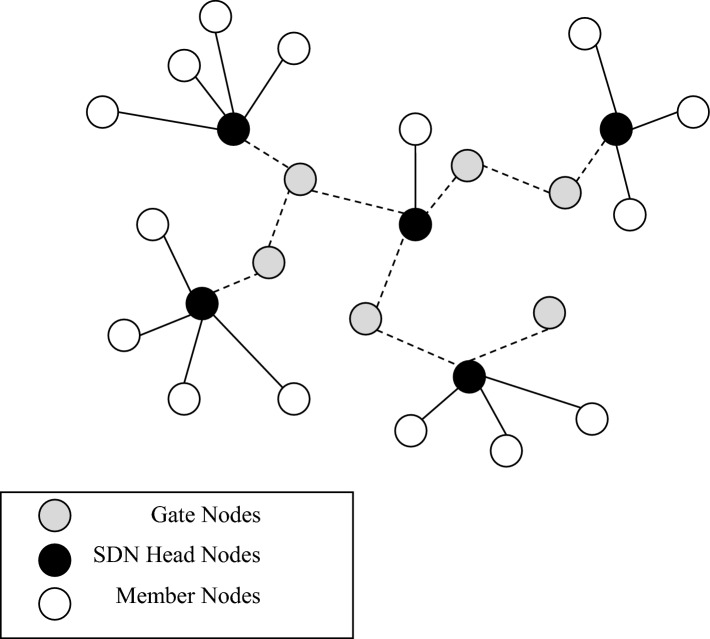
A view of the topology structure formed in the proposed method.

Considering this feature, the network clustering structure will become a hierarchical tree topology, an example of which is shown in Fig. 5.

In Fig. 5, each SDN controller and its neighboring nodes are considered as a sub-domain in SDN. Communication between subdomains is facilitated through gateway nodes (shown in gray in the figure). Additionally, the connections between the controllers are illustrated with dashed lines in this figure. Once each cluster is formed, the controller node will possess its member list and exchange it with other controllers. After establishing the network topology, route selection, and data exchange operations will be executed. This step of the proposed method will be further elucidated in the following section.

#### Data routing using a structured structure

After forming the network topology, this structure will be utilized for data routing concerning library information. Due to the tree topology, it is evident that there will be only one path between each controller node and the data center. Thus, if a node intends to send a request to receive information from the data center, the source node initially transmits its ID to its subdomain controller. The controller node then searches for the source ID in its member list, and if it corresponds, it forwards the sensor request to the central node through the unique path in the hierarchical tree. Upon receiving this message, the central node matches the requester's sensor ID with the requested information ID, and if the information is accurate, the requested media is transmitted to the controller node through the existing path.

## Result and discussion

The approach under consideration underwent a thorough assessment comprising two specific stages. During the initial phase, we concentrated on the practical application in a small-scale library for a month. Through this on-site evaluation, we scrupulously examined the effectiveness of the suggested architectural method, making comparisons with the library's conventional approach during the same period. In the subsequent phase, we created a simulation environment utilizing MATLAB 2021. In this stage, we methodically evaluated the performance of the proposed algorithm, offering a detailed analysis in contrast to the algorithm outlined in the reference articles^25^ and^6^.

### Real-world implementation results

**Figure 6:**
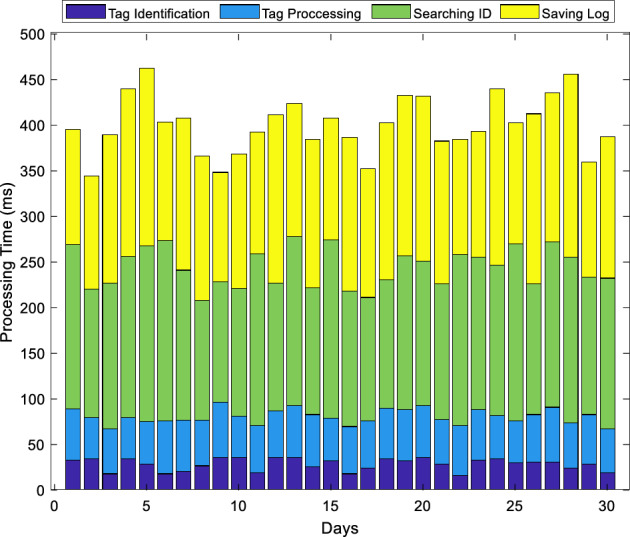
The average processing time of the proposed system for recording the transaction of library items.

**Figure 7:**
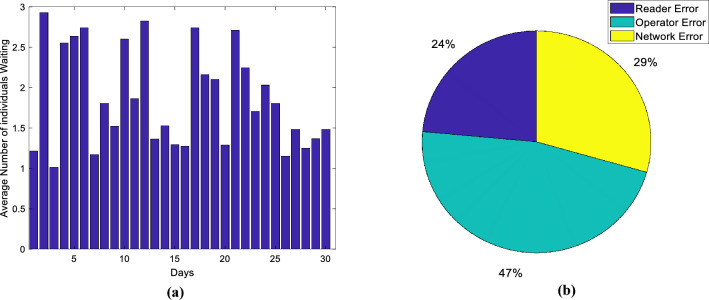
System performance chart in transaction registration (**a**) average accuracy per day, (**b**) frequency of error types.

**Figure 8:**
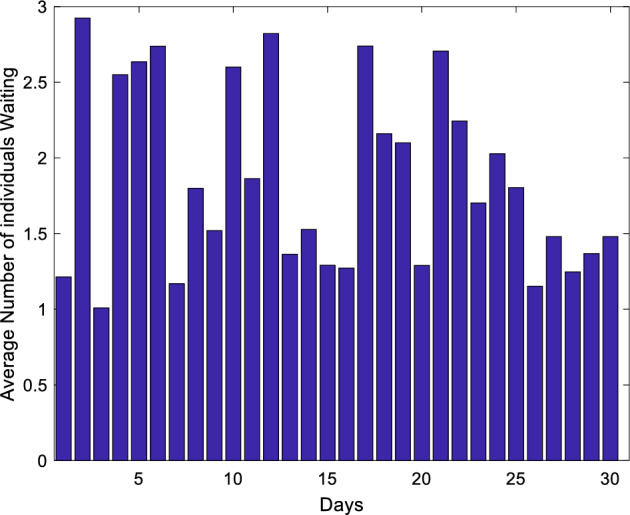
Average demand queue by day.

**Figure 9:**
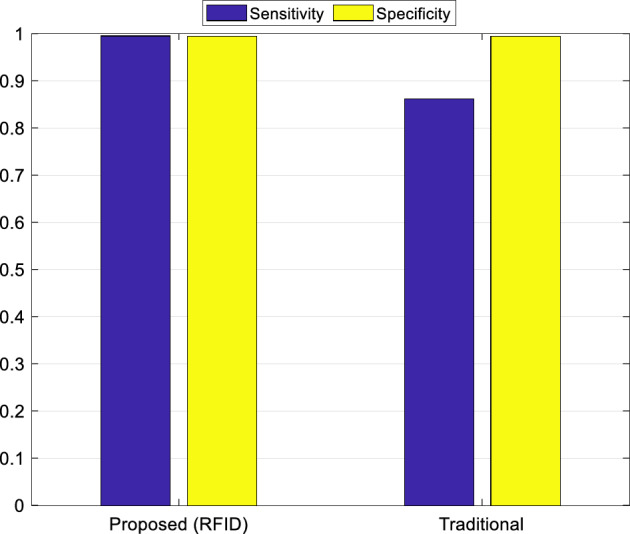
Comparison of sensitivity and specificity criteria of the proposed method with the traditional method.

The proposed architecture addresses the evolving landscape of smart libraries by introducing an IoT-based low-cost system leveraging SDN. Targeting RFID-based processes, such as authentication, property circulation, and book loans, the system intelligently oversees various library activities, reducing the need for constant operator supervision and providing scalability for extensive library environments. The evaluation of this architecture over a one-month period, as indicated in Figs. 6,7,8,9, demonstrates its practical implementation in a small-scale library, highlighting its potential to enhance efficiency and cost-effectiveness in managing library processes. Figures 6,7,8,9 pertain to the first phase, where we conducted evaluations at various instances to record library item transactions, encompassing four components: the first involves tag identification, the second encompasses tag processing, the third engages in ID searching, and the fourth involves saving the results. Average processing times were scrutinized at different intervals, revealing that, on the whole, this architecture can accomplish each transaction in under half a second, signifying a substantial enhancement in system speed. Figure 7 illustrates accuracy and the frequency of error types. The average accuracy rate is presented in Fig. 7.a, calculated by determining the error count on different days, dividing it by the total number of transactions, and deriving its rate. The results, as depicted in Fig. 7.a, indicate that the proposed method attains a minimum accuracy of 99% on various days, demonstrating an error probability of less than one percent. Figure 7b further categorizes errors by type, revealing three categories: (1) tag reader error, (2) user error, and (3) network error, with user error exhibiting the highest error rate. Nearly half of the errors are attributed to user operator mistakes, largely attributed to users' unfamiliarity with the system, although this has significantly decreased over different days with the operators gaining more familiarity with the system.

Figure 8 presents the daily average number of demand queues, determined over a span of days, with an observed average of 1.4 queues. This signifies that, even in the presence of continuous demand within the library system, the number of individuals in the demand queue remains below a specified threshold, effectively preventing congestion in the system.

In Fig. 9, a comparative analysis is conducted between the proposed smart system, denoted as RFID, and the traditional user-based system, focusing on specificity and sensitivity criteria. This comparison assesses whether the RFID system excels in identifying users and making book judgments compared to human users. The findings suggest that the proposed method, marked as RFID, demonstrates relatively high accuracy and ideal performance compared to the traditional system.

### Simulation results

**Figure 10:**
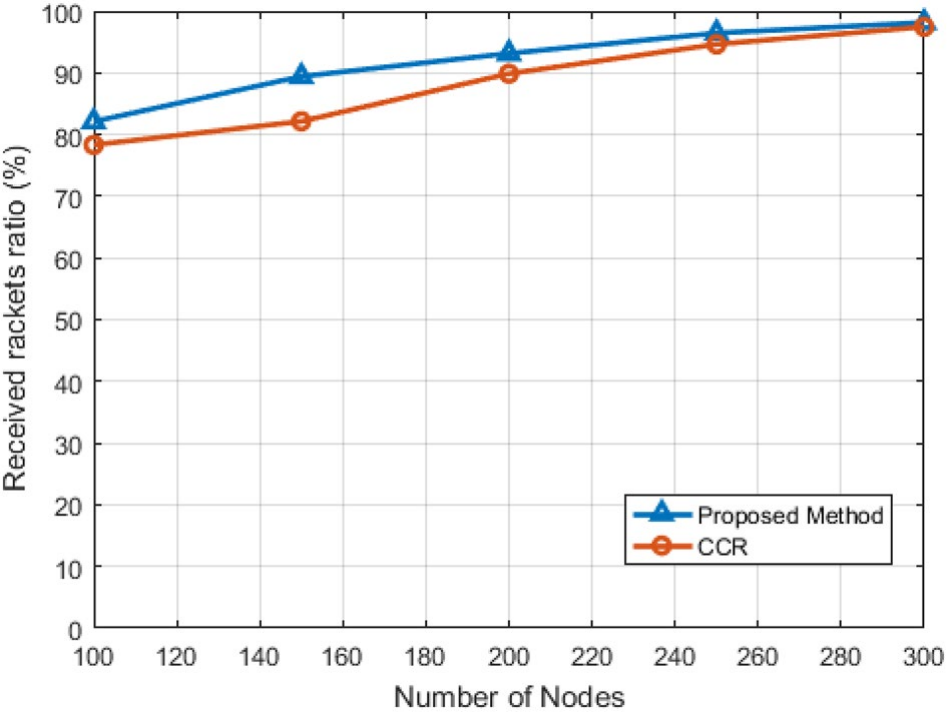
The percentage of successful reception of packets against the number of visiting nodes.

**Figure 11:**
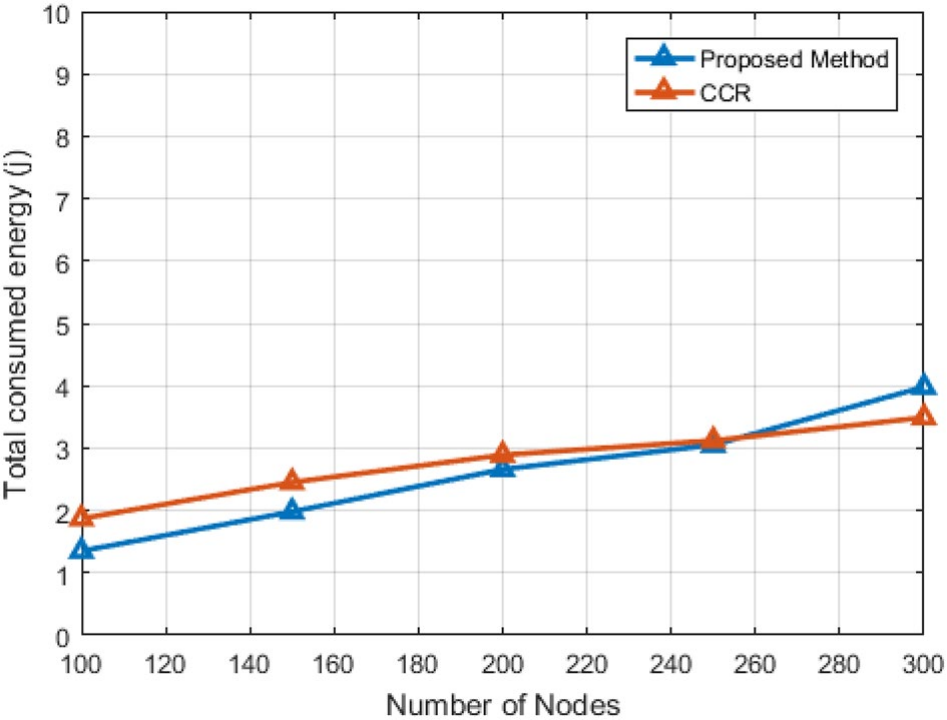
Energy consumption of the whole network in relation to the number of visiting nodes.

**Figure 12:**
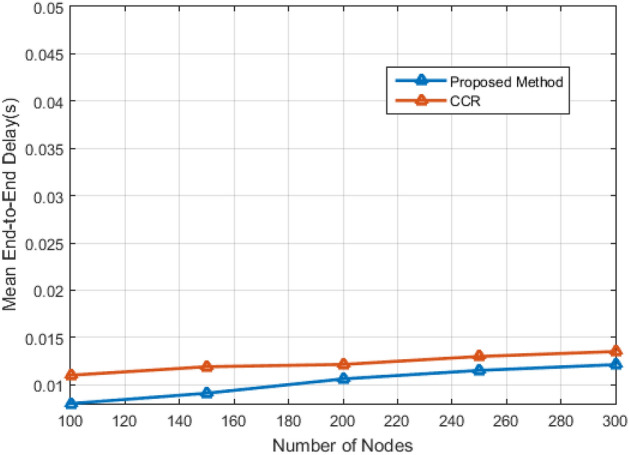
Average end-to-end network latency versus number of visitors.

**Figure 13:**
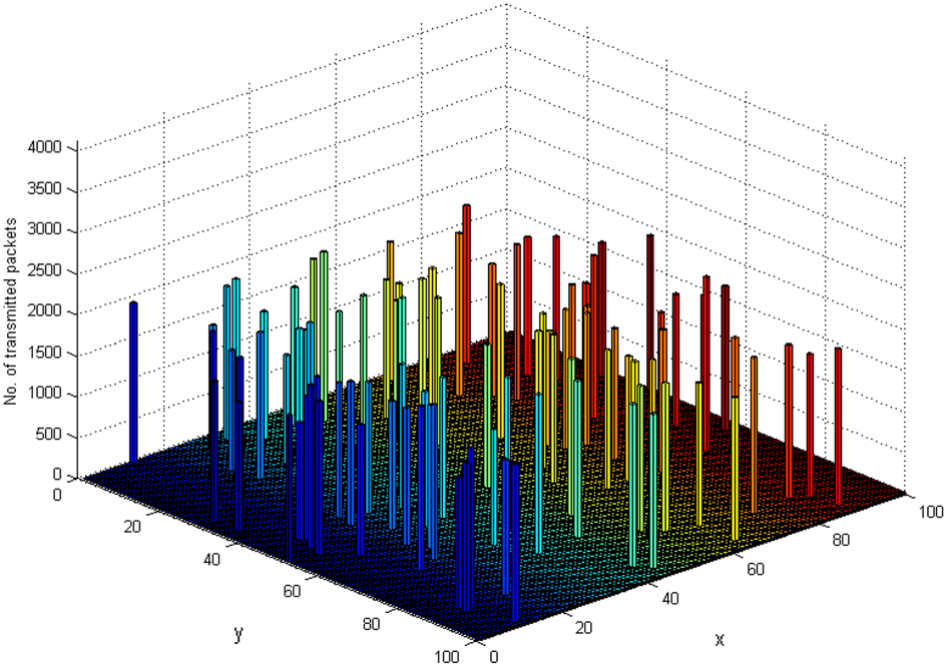
Load imposed on each node for forwarding data according to the position of that node in the environment.

In this operational phase, where the proposed method is simulated in an environment, we have focused on the construction of topology and information routing between the nodes of the smart library network and compared the results with^6^ and^25^. Moving on to Figs. 10,11,12,13, these figures delve into a comprehensive comparison of the proposed method with previous approaches. Figure 10 specifically addresses the packet delivery rate concerning changes in the number of network nodes. Through a simulated environment involving 100 to 300 nodes with heterogeneous characteristics, the figure reveals an increasing packet delivery rate as the number of nodes rises. This is attributed to the multi-step method employed, utilizing users themselves to exchange data, resulting in a higher probability of successful packet delivery.

Figure 11 shifts the focus to energy consumption, indicating an exponential increase in constant energy as the number of nodes rises. Interestingly, the proposed method consumes less energy up to 250 nodes, exceeding the compared method for 300 nodes. This lower energy consumption for 100 to 250 nodes indicates superior performance in terms of energy efficiency, attributed to the use of an efficient energy topology in the software network architecture considered. However, as the number of nodes reaches 300, energy consumption rises due to significant differences in transmissions between the proposed and compared methods.

Figure 12 calculates the average user delay, revealing consistently lower delays in the proposed method across various scenarios. Lastly, Fig. 13 showcases the successful load balancing achieved by the proposed method. The equal participation of nodes in sending data, regardless of their position, demonstrates the method's effectiveness in load distribution, contributing to an extended network lifespan and enhanced efficiency.

## Conclusion

Our research paper proposes an innovative IoT-based low-cost architecture for smart libraries using SDN, presenting a comprehensive method for imbuing libraries with intelligence. The core contribution lies in the synergy of IoT and SDN technologies, showcasing the capacity to intelligently oversee diverse library activities, thus reducing the need for constant operator supervision. Our proposed framework incorporates RFID-based processes for authentication, property circulation management, and library book loan management, while the data exchange relies on IoT SDN. The architecture encompasses key components such as a data center, SDN controllers, RFID tags, tag readers, and other network sensors. Our proposed architecture offers a transformative approach to traditional libraries, enhancing key processes through intelligent information management. The data center plays a pivotal role in organizing crucial information related to books, members, and book loans, facilitating efficient library resource management. Leveraging RFID technology for member and book identification, the proposed system ensures secure and streamlined processes. The integration of SDN-based network configuration involves a hierarchical tree topology and a weight-based algorithm for efficient cluster connectivity, addressing concerns related to cost and scalability. Our innovative approach provides a foundation for modernizing extensive libraries and lays the groundwork for future advancements in the integration of IoT and SDN technologies.

## Data Availability

All data generated or analysed during this study are included in this published article.
